# Differential Roles of HOW in Male and Female *Drosophila* Germline Differentiation

**DOI:** 10.1371/journal.pone.0028508

**Published:** 2011-12-06

**Authors:** Adrian C. Monk, Nicole A. Siddall, Barbara Fraser, Eileen A. McLaughlin, Gary R. Hime

**Affiliations:** 1 Department of Anatomy and Cell Biology, University of Melbourne, Parkville, Victoria, Australia; 2 School of Environmental and Life Sciences, University of Newcastle, Callaghan, New South Wales, Australia; University of Otago, New Zealand

## Abstract

The adult gonads in both male and female *Drosophila melanogaster* produce gametes that originate from a regenerative pool of germline stem cells (GSCs). The differentiation programme that produces gametes must be co-ordinated with GSC maintenance and proliferation in order to regulate tissue regeneration. The HOW RNA-binding protein has been shown to maintain mitotic progression of male GSCs and their daughters by maintenance of Cyclin B expression as well as suppressing accumulation of the differentiation factor Bam. Loss of HOW function in the male germline results in loss of GSCs due to a delay in G2 and subsequent apoptosis. Here we show that female *how* mutant GSCs do not have any cell cycle defects although HOW continues to bind *bam* mRNA and suppress Bam expression. The role of HOW in suppressing germ cell Bam expression appears to be conserved between sexes, leading to different cellular outcomes in *how* mutants due to the different functions of Bam. In addition the role in maintaining Cyclin B expression has not been conserved so female *how* GSCs differentiate rather than arrest.

## Introduction

**Figure 1:**
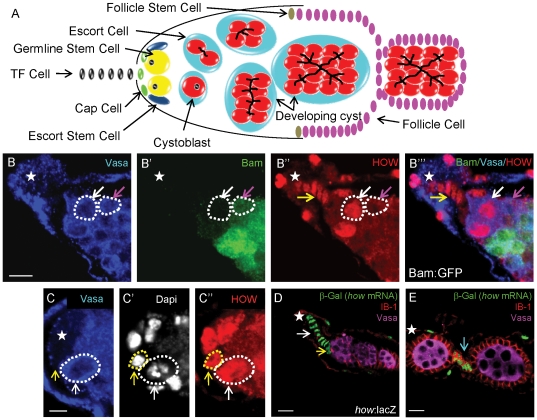
HOW is expressed in the early female germline. (**A**) schematic of the anterior region of an ovariole. GSCs (yellow) are anchored to Cap Cells (green). GSCs divide asymmetrically to produce one self-renewed daughter stem cell (yellow), and one cystoblast (red). The cystoblast divides four times to produce cysts of 16 interconnected cells. Escort stem cells (dark blue) are also in contact with cap cells and generate escort cells (light blue), while follicle stem cells (brown) produce follicle cells (magenta) and stalk cells. (**B**) Anti-HOW (red) labels GSCs (white arrow) and cystoblasts. HOW levels are reduced by the 2-cell stage (magenta ↑) when Bam expression (green) is first detected (*bam*::GFP reporter). HOW is present in the terminal filament (TF) cells (yellow ↑), and (**C**) in cap cells (yellow ↑) adjacent to Vasa-positive GSCs (white ↑). (**D,E**) A β-Galactosidase stain on a *how*:lacZ enhancer trap shows presence of lacZ in the somatic cells of the ovary, including terminal filament cells (white ↑), cap cells (yellow ↑), and stalk cells (blue ↑). Scale bar 5 µm for B,D,E, 2 µm for C. Anterior marked (*).

Tight regulation of stem cell differentiation is crucial for maintaining tissue homeostasis in all stem cell niches. The balance between proliferation and differentiation must be delicately maintained in order to prevent cell depletion or formation of undifferentiated neoplasms. The *Drosophila* germline acts as a good model to study germ cell regulation, as both male and female reproductive organs contain germline stem cells (GSCs) in a tightly regulated niche. The adult testis usually contains 9–10 GSCs surrounding somatic hub cells [1], while adult ovaries typically contain 2–3 GSCs associated with 4–7 neighboring somatic cap cells [2] (Figure 1A). GSCs are physically attached to their supporting somatic niche cells via cadherin based connections [3]. Both niches are responsible for providing localized proliferative signals to maintain stem cell identity and prevent premature differentiation. Maintenance signals are very specific and are believed to span only one cell diameter [2]. In order to produce a gamete, both populations of GSCs divide asymmetrically to produce a daughter GSC and one daughter cell displaced away from the niche, which begins its commitment toward differentiation. This daughter cell, the gonialblast in the male and cystoblast in the female, begins mitotic amplification with incomplete cytokinesis to produce a cyst of 16 interconnected proliferative cells. In both sexes, mitosis ceases at this point and the two germ cell populations differentiate in very different ways to eventually produce mature sperm or an oocyte. In addition to GSCs, both organs contain another somatic stem cell population that is in contact with the niche and GSCs. In males, cyst stem cells (CySCs) divide in coordination with GSCs to produce cyst cells, which encapsulate the dividing spermatogonial cells, while in females escort stem cells (ESCs) produce escort cells, which perform an analogous role in the ovary. Cyst stem cells also form an important component of the male GSC niche.

Regulation of GSC differentiation in the testis and ovary share common signals, however their action in the respective organs is sometimes very different. Differentiation of GSCs in both sexes is prevented by the Jak/STAT and BMP signaling pathways, however their specific mechanism of action is sex-specific. In the female germline, BMP signaling maintains GSC identity [4]. The ligands Decapentaplegic (Dpp) and Glass Bottom Boat (Gbb) are secreted primarily from cap cells and act directly on GSCs to repress the major differentiation-promoting gene *bag of marbles* (*bam*), by binding to silencer elements in the *bam* gene, thus preventing transcription [5], [6]. This repression is relieved in the cystoblast allowing *bam* transcription, which together with its partner *benign gonial cell neoplasm* (*bgcn*), acts to commence cystoblast differentiation.

In the male germline, there is also a requirement for BMP signaling, however it is slightly different than in the female germline. Gbb is produced by the somatic hub and cyst cells, and is required to repress *bam* transcription [7], [8]. However, despite being required for GSC maintenance, BMP signaling is not sufficient to specify GSC fate directly, as in the female germline. This role may be fulfilled by the Jak/STAT signaling pathway in males [2]. Here, Jak/STAT signaling is required for GSC self-renewal [9], [10]. Somatic hub cells in the testis secrete the ligand Unpaired (Upd), which activates the Jak/STAT pathway in CySCs[11]. This leads to expression of the transcriptional repressor Zfh-1, and maintenance of CySCs in an undifferentiated state, allowing for continued BMP-mediated *bam* repression in GSCs [11]. In the female germline, there appears to be no autonomous requirement for Jak/STAT signaling in GSCs, however it has recently been shown that STAT is required in ESCs for their maintenance [12]. Hence, despite the conservation of genes used in both systems, how they exert their effect on stem cell self-renewal is very different.

The use of these signaling pathways in different ways may, in part, be due to the sex-specific roles of the key differentiation-promoting gene *bam* [13], [14]. In males, Bam is first detectable in 4-cell spermatogonia and levels accumulate to a threshold amount, thereby initiating differentiation of 16-cell clusters [15]. High levels of Bam bring about an earlier onset of spermatogonial differentiation whereby cells differentiate before all four rounds of proliferation are complete [15]. Conversely, low levels of Bam result in a longer time required to reach the threshold necessary for spermatogonial differentiation, resulting in spermatogonia undergoing extra rounds of mitosis generating tumorous cysts of proliferative undifferentiated cells [15].

In females, Bam is required in one daughter of the GSC asymmetric division, to specify the cystoblast-fate [16]. Forced Bam expression in stem cells leads to premature differentiation of GSCs into cystoblasts [17], while low levels of Bam result in a halt to the differentiation pathway, and both GSC daughters generated from asymmetric division continue to proliferate, generating large numbers of GSC-like cells [16]. The amount of Bam protein required to initiate differentiation must be very low in cystoblasts, as it is undetectable by immunostaining or reporter gene activity until the 2-cell stage. This is in stark contrast to the male germline, where levels are relatively high and must reach a threshold in order to promote differentiation [15].

Recently a number of other factors have been identified which are required to regulate *bam* expression including ISWI [18], involved in chromatin remodeling, Otefin [19], a nuclear membrane protein, EIF4A [20], a translational initiation factor, and Piwi [21], a nucleoplasmic protein present in both somatic and germline cells [22]. This level of control indicates the importance of tight regulation on key developmental regulators. We have also identified that the RNA-binding protein Held-Out Wings (HOW) is also important for Bam regulation in the male germline [23]. HOW has previously been shown to act post-transcriptionally to regulate translation of target mRNAs [24]. We showed that *bam* mRNA is regulated in GSCs and gonialblasts by How(L), a predominantly nuclear HOW isoform and a demonstrated repressor of mRNA expression [25]. Increased levels of How(L) resulted in a delay to the Bam expression domain, and consequently, the differentiation from spermatogonia to spermatocyte was delayed, and cells continued to proliferate beyond their normal four rounds of mitotic amplification. Conversely, loss of HOW resulted in premature differentiation of spermatogonia, with cysts of spermatocytes observed containing eight cells, indicating that these cells only completed three rounds of mitotic amplification, prior to the Bam differentiation threshold being reached.

We also observed an additional role for HOW in the male germline, presumably unrelated to suppression of *bam*. HOW was required for GSC maintenance, and GSCs lacking HOW function were lost very quickly from the niche via apoptosis. We showed that there was an indirect interaction between *how* and *cyclinB* (*cycB*) in the male germline. CycB has been shown to be absolutely required for germ cell mitoses in the germline [26]. Loss of *how* led to low levels of CycB in germ cells, resulting in a delayed G2 phase of the cell cycle as cells were unable to enter mitosis, leading to cells growing abnormally large, and eventually removed from the germline via cell death. High levels of How(L) led to a faster G2 phase as cells were unable to downregulate CycB at the normal time points. HOW is therefore required for maintaining CycB levels in GSCs and spermatogonia.

In this study we show that HOW is also required in the female germline for GSC maintenance, as GSCs lacking HOW are lost from the niche. The HOW expression pattern in the female germline was found to be slightly different to that in the male germline, which reflects the differential expression pattern of Bam in females. The exact inverse staining pattern has been conserved however, with levels of HOW downregulated at the stage when Bam is first detectable. Unlike in the male germline, we observed that *how* does not regulate *cycB* in the female germline, and hence is uncoupled with transit amplifying divisions. However, we did observe an interaction with *how* and *bam* in the female germline. HOW binds *bam* mRNA and ectopic expression of How(L) resulted in a delay in the accumulation of Bam protein in cystoblasts, and hence more GSCs were observed in these germaria. This phenotype resembled what has been observed previously in *bam* heterozygote germaria [20]. Thus, we believe that, as in the male germline, *how* is responsible for post-transcriptional regulation of *bam* mRNA in the female germline. Unlike in the male, the GSC loss observed in *how* germaria is associated with *bam* deregulation, rather than suppression of CycB in observed in *how* testes.

## Results

### HOW is expressed in the early female germline

To determine the expression pattern of HOW in the female germline, we used a specific polyclonal α-HOW antibody [25], and immunostained ovaries from adult flies carrying the *bam::GFP* transgene [27]. Germaria from this genotype appear phenotypically normal and express *bam*-driven GFP from the 2-cell stage to the 16-cell stage (Figure 1B'). Similar to the male germline, HOW protein was detected in Vasa-expressing germline stem cells in the female germline, as well as in the cystoblast, the female equivalent of the gonialblast (Figure 1B”). However, HOW expression was downregulated by the 2-cell stage in the female germarium (Fig 1B”), which is spatially more constricted than in the testis where HOW is detected in 2 cell cysts. In the germarium, *bam::GFP* is first detected one cell division earlier (2-cell stage) than in the male germline (4-cell stage), making HOW expression complimentary to *bam::GFP* in both the male and female germline despite slight expression pattern differences. HOW expression in the germline appeared predominantly nuclear, again suggesting the prevalence of the nuclear HOW isoform, How(L) (Fig 1B’’, C’’).

HOW protein was also detected in some somatic cells of the ovary, including terminal filament cells (Figure 1B’’), cap cells (Figure 1C), and stalk cells. We also analysed ovaries from a *how*:lacZ enhancer trap (Bloomington #12151). β-Galactosidase (indicating the presence of *how* mRNA) was also detected in the terminal filament cells and cap cells (Figure 1D), as well as stalk cells (Figure 1E), however it could not detected in the germline. This suggests that the P-element enhancer trap did not respond to germ cell enhancers or they have been disrupted.

### HOW is required intrinsically for ovarian GSC maintenance

**Figure 2:**
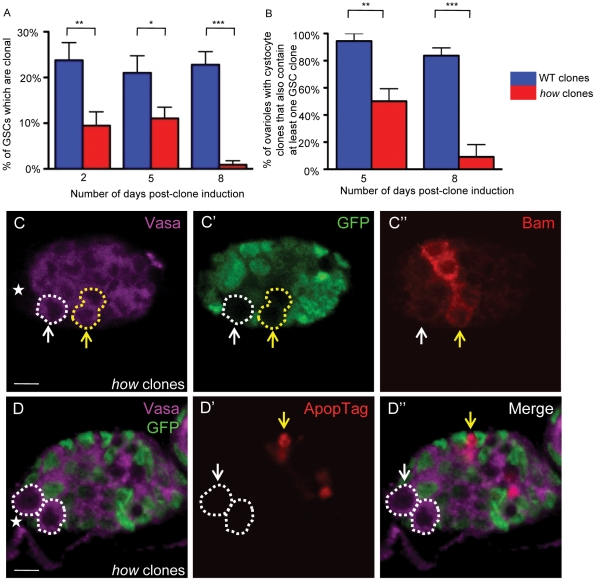
HOW is required intrinsically by ovarian GSCs for their maintenance. (**A**) Comparison of wild type and *how* ^stru^ GSC clone maintenance over time at 2 (p = 0.005), 5 (p = 0.02), and 8 days (p<0.0001) post-clone induction, indicates that GSCs lacking HOW are lost from the niche over time. (**B**) *how* ^stru^ clonal cystocytes persist longer than *how* ^stru^ GSC clones. 94% (n = 18) of wild type cystocyte clones five days and 84% (n = 43) eight days post-clone induction also contain a parental GSC clone. In ovaries containing *how* ^stru^ cystocyte clones, only 50% (n = 30) at 5 five days and 9% (n = 11) at 8 days post-clone induction still possess a parental GSC clone, indicating *how* cystocytes derived from *how* clonal GSCs lose their progenitor GSC over time (p = 0.001 at 5 days and p<0.0001 at 8 days). (**C**) *how* ^stru^ GSC clones (white ↑) do not express detectable levels of the differentiation marker Bam (red). *how* clonal cystocytes (yellow ↑) derived from *how* GSCs express Bam at normal levels and timing. (**D**) A germaria containing ApopTag-negative *how* GSCs (white dotted line) with a dying IGS cell (yellow ↑). Scale bar 5 µm. Anterior marked (*).

To specifically investigate the function of HOW in the female germline, we induced homozygous mutant clones carrying the strong LOF allele *how^stru-3R-3^* [28] and compared these directly to wild type clones at various time points post-clone induction. Previously, we showed that male *how* GSC clones do not display detectable levels of HOW protein using immunostaining [23]. Two days after heat-shock induction, control GSC clones were present at a frequency of 24% (n = 122), whilst homozygous *how^stru^* GSC clones were present at a much lower frequency (9% GSC clones observed, n = 95). Although control GSC clones were maintained after clone induction (5 days 21%, n = 119, and 8 days 23%, n = 215), *how* GSC clones were rapidly lost. At 8 days only 1% of GSCs counted were *how* clones, n = 112 (Figure 2A). Unlike in the male germline, GSCs can survive for a short time without *how* function, however these are ultimately lost from the GSC niche. This may indicate that in the female germline, *how* may not be required for cell survival but for prevention of differentiation. These experiments provide support for an intrinsic role for HOW in the division or maintenance of GSC identity. In the female germline, Bam is required for the cystoblast-fate during asymmetric stem cell division; however the protein is not observable until the 2-cell stage. If *how* GSCs were being lost due to premature differentiation, an elevation in Bam levels may be present in mutant GSCs but *how* GSC clones did not show detectable levels of Bam (Figure 2C). This does not rule out the possibility that *how* GSCs are lost due to premature differentiation, however, as it has been shown that Bam can initiate cystoblast differentiation despite protein levels not being observable in the cystoblast, suggesting that levels of Bam required for differentiation must be very low.

The process of GSC division through to egg chamber formation occurs over a 7 day period at 25°C. Hence, at eight days post clone induction, any cystocyte clones observed in the germarium are assumed to be derived from clonal GSCs, since any clones that were initiated in post-GSC germ cells would normally have progressed to the egg chamber region. At five days post clone induction, 94% (n = 18) of ovaries that contained wild type cystocyte clones in the germarium also had at least one GSC clone, which is as expected since a GSC clone not only self-renews but also produces a cystoblast that is committed to differentiate (Figure 2B). However, in ovaries containing *how^stru^* mutant clones in the germarium, only 50% (n = 30) still possessed a progenitor GSC clone after five days. At eight days, 84% (n = 43) of ovaries with wild type cystocytes clones still possessed a GSC clone, while just 9% (n = 11) of ovaries containing *how* cystocyte clones still possessed a parental clonal GSC. This indicates that GSCs were more sensitive to the loss of HOW function and were rapidly lost from the niche due to premature differentiation. In contrast, cystocyte clones persisted for a longer period of time, also suggesting that HOW is not required for cell survival. In the male germline, *how* germ cells arrested at the 2-cell stage due to a G2 cell cycle defect and were eliminated via apoptosis, however this was not observed in the female germline (see below), again indicating that female *how* germ cells were prematurely differentiating.

To determine whether daughter cells derived from *how* clonal GSCs were able to differentiate into 2-cell clones, we examined Bam levels in *how* cystocytes. Bam is normally detectable from the 2-cell stage [16], and we observed detectable levels in *how* cystocytes (Figure 2C), indicating that the progeny of *how* GSCs can differentiate normally into cystocytes.

In order to investigate the possibility that *how* GSCs were being lost from the niche via apoptosis, we performed the ApopTag cell death assay on germaria containing wild type and *how* GSC clones (Figure 2D). We observed zero Apoptag-positive GSCs in wild type GSC clones (n = 21), although occasional cells in later cysts were observed to apoptose (Figure 2D), or *how* GSC clones (n = 23), indicating that *how* GSCs are not being lost via apoptosis.

### HOW does not affect germ cell mitoses in the female germline

**Figure 3:**
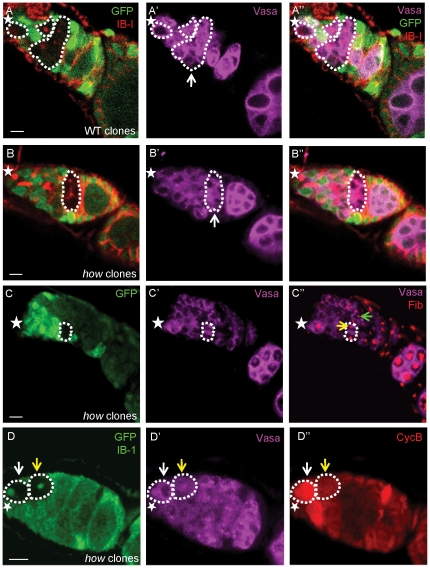
HOW is not required for germ cell mitoses in the female germline. (**A,B**) Comparison of germaria containing wild type germ cell clones (A, white dotted line) and *how* ^stru^ germ cell clones (B, white dotted line). (**A**) Five days post-clone induction, wild type clones derived from wild type GSC clones have reached the 16-cell stage (white ↑). (**B**) *how* ^stru^ germ cell clones derived from *how* ^stru^ GSC clones progress to the 16-cell stage (white ↑), as seen by the branched fusome (red) connecting Vasa-positive (magenta) clonal germ cells. (**C**) Germ cells lacking HOW do not show morphological defects. Five days post-clone induction, *how* clonal cystocytes can be observed (white dotted line). (**C’’**) Anti-Fibrillarin stains the nucleolus (red). Nucleolar size in *how* clonal cystocytes (yellow ↑) is similar in size compared to the nucleoli in control cystocytes at a similar stage in development (green ↑). (**D**) Female germ cells lacking HOW are able to produce CycB protein. Wild type (non-clonal, GFP-positive) GSCs and cystocytes up to the 8-cell stage display oscillating levels of CycB. (**D**'–**D’’**) *how* GSCs (white ↑) and cystocytes (yellow ↑) can express CycB (red) at normal oscillating levels. Anterior marked (*). Scale bar 5 µm.

Spermatogonia derived from *how* GSCs in the male germline did not undergo the normal mitotic divisions and stalled at the two-cell stage subsequent to loss by apoptosis. To determine if a similar defect was present in cystocytes derived from female *how* GSCs, *how* ^stru^ GSC clones were generated and the progress of clonal germ cells derived from these cells was followed. At 5 days post clone induction, wild type germ cell clones were observed at each of the 2, 4, 8 and 16 cell stages (Figure 3A). In ovaries containing *how* germ cell clones, while the parental GSC had often been lost, derived clonal germ cells from this parental GSC did not stall at the 2-cell stage. Cystocytes lacking HOW were able to progress to the 16-cell stage (Figure 3B). This indicates that, unlike in males, loss of HOW in females does not result in a stalled cell cycle, and may indicate HOW plays a different role in the female germline. Additionally, *how* cystocytes did not appear to carry any obvious morphological defects. In males, *how* spermatocytes showed numerous defects including increased nucleolar size, indicative of increased ribosome biogenesis [23]. In cystocytes lacking HOW, nucleolar size was comparable to nucleoli in neighboring wild type cystocytes at the same stage of development (Figure 3C).

As male germ cells lacking HOW failed to complete mitotic divisions due to a lack of CycB protein, we investigated whether loss of HOW in female germ cells had any effects on CycB. As *how* cystocytes did not show mitotic defects, *how* cystocytes should synthesize CycB at levels similar to wild type cystocytes. By inducing *how* GSC clones and dissecting ovaries 7 days post-heat shock, we observed that *how* germ cells were able to synthesize CycB (Figure 3D). Similar to male germ cells, levels of CycB in female germ cells oscillate between high and low levels during the cell cycle, peaking prior to M phase in order to initiate mitosis. Both *how* GSCs and *how* cystocytes had detectable levels of CycB. 27% of *how* clonal germ cells counted (n = 56) were CycB positive, compared to 33% (n = 72) for wild type twin-spot control clones (p = 0.4, ns). In male *how* germ cells, CycB was completely absent in the majority of germ cells, however in female *how* germ cells, complete absence of CycB was never observed. These data indicate that *how* germ cells are able to synthesize and degrade CycB, unlike in the male germline. This shows that HOW is playing a different role in males to females. In males, HOW appears to regulate TA divisions via suppression of *bam* expression but is also required for germ cell mitoses, whereas in females, HOW does not appear to affect the cell cycle.

### Overexpression of HOW(L) causes extra GSC-like cells

**Figure 4:**
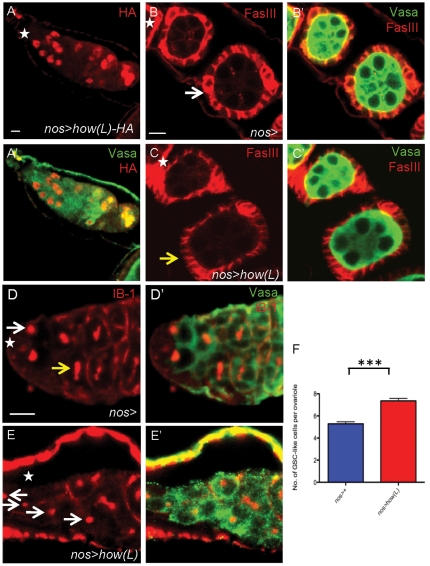
Overexpression of HOW(L) in the female germline results in extra GsC-like cells. (**A**) The *UAS:HOW(L)* construct contains an HA-tag. Anti-HA staining (red) on *nos>how(L)* germaria shows that expression of this transgene is beyond the normal HOW expression domain. (**B,C**) Ectopic HOW(L) does not result in extra rounds of germ cell mitoses**.** (**B**) In control ovaries (*nos>w* ^1118^) a cystoblast will undergo four rounds of mitosis to generate 16 interconnected cystocytes. These develop into egg chambers containing one oocyte and 15 nurse cells (green), surrounded by follicle cells (red). (**C**) Overexpression of HOW(L) in the female germline results in egg chambers which contain exactly 16 cells (an oocyte and 15 nurse cells). (**D**–**F**) Overexpression of HOW(L) results in germaria containing more GSC-like cells (**E,E**') than in control germaria (**D,D**')**,** as observed by germ cells (green) displaying a round spectrosome (red, white ↑), not a branched fusome (yellow ↑). Anterior direction marked (*). Scale bar 5 µm.

To determine what effect high levels of HOW had on the female germline, we overexpressed HOW(L) from p*UAST*:*how(L)* [25] in early germ cells using the *nos*:Gal4 driver [29]. This vector has previously been used to generate phenotypes in the female germline [30], [31]. Our transgene was tagged with an HA-tag, and immunostaining using an anti-HA antibody revealed that raising flies at 29°C allowed transgene expression using the *nos*:Gal4 driver in the progeny of GSCs (Figure 4A). In the male germline, expression of HOW(L) resulted in germ cells undergoing extra rounds of spermatogonial mitosis. To determine whether HOW was acting in a similar manner in females as in males, we investigated whether ectopic HOW(L) resulted in extra germ cell mitoses. In wild type germaria, a cystoblast undergoes four rounds of mitosis to generate 16 germ cells, which form 15 nurse cells and one oocyte (Figure 4B). These can be easily counted by examining developing egg chambers. Overexpression of HOW(L) in the germline (*nos>how(L)*) did not result in female germ cells undergoing extra rounds of mitotic divisions (Figure 4C). 100% of egg chambers counted contained 16 germ cells (n = 23). Therefore, unlike in males, overexpression of HOW(L) in the female germline did not result in extra cystocyte mitotic divisions.

As *bam* plays a different role in the female germline compared to the male germline, this wasn't an unexpected result. *bam* is required in females to ensure one stem cell daughter cell differentiates into a cystoblast, therefore if HOW(L) is repressing *bam* in the female germline, we postulated that *nos>how(L)* germaria would contain extra GSCs, as it has previously been shown that loss of *bam* results in extra GSCs [16]. In order to quantify this, we counted the number of Vasa-positive germ cells with a spectrosome (not a branched fusome), and termed these cells “GSC-like” cells, as it was not possible to accurately distinguish between GSCs and their direct daughter cells. Therefore, we compared the number of GSC-like cells in *nos>how(L)* germaria to *nos*:Gal4 control germaria. In control germaria, the average number of GSC-like cells was 5.3+0.2 (n = 25) (Figure 4D, F). Overexpression of HOW(L) in the female germline resulted in an increased number of GSC-like cells (Figure 4E). *nos>how(L)* germaria contained 7.4+0.2 (n = 28) GSC-like cells per ovariole (Figure 4F), indicating that overexpression of HOW(L) resulted in a mild increase in GSC-like cells.

This increase may be a milder version of the phenotype observed in *bam* ovaries, which produce “GSC-tumors” [16]. As the *nos>how(L)* phenotype was similar to *bam*/+ heterozygotes in males, we performed the same experiment on *bam*/+ heterozygous ovaries. *bam*/+ ovaries contained 7.6+0.2 (n = 30) GSC-like cells per germaria, indicating that *bam* heterozygous ovaries also show a mild increase in GSC-like cell number, but do not form GSC tumors. Therefore, the *nos>how(L)* phenotype resembles the *bam/*+phenotype, and suggests that HOW(L) may be repressing *bam* in the female germline as well as the male germline.

### *how* genetically interacts with *bam* in the female germline

**Figure 5:**
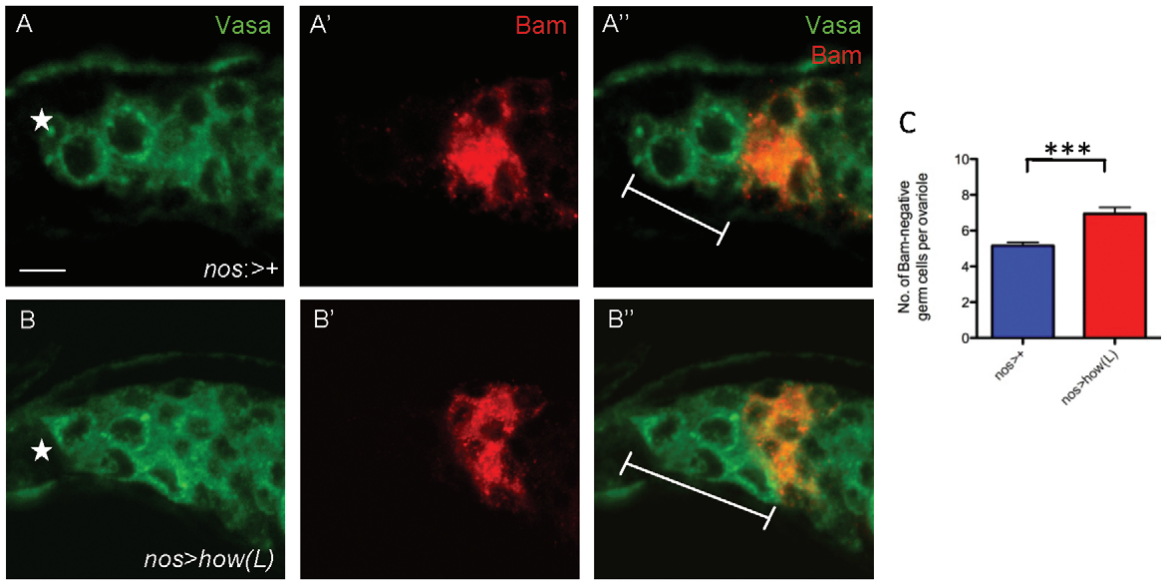
Overexpression of HOW(L) delays the expression of Bam. Comparison of the onset of Bam accumulation in ovaries. (**A**) In control ovaries *(nos:*Gal4), 2–3 GSCs reside at the anterior position of the germarium. Bam (red) is first detectable in 2-cell cystocytes. Therefore, GSCs and cystoblasts are Bam-negative in the region anterior to Bam expression (white line) in wild type germaria. (**B**) Overexpression of HOW(L) in the female germline (*nos>how(L)*) results in more early germ cells in the region anterior to Bam expression. (**C**) Statistical analysis showing the number of early germ cells in *nos>how(L)* germaria is greater than *nos*:Gal4 ovarioles (p<0.0001). Anterior marked (*). Scale bar 5 µm.

In the male germline when HOW(L) was ectopically expressed, a delay in the expression pattern of Bam antibody staining was observed. We therefore examined whether overexpression of HOW(L) in the female germline also had an effect on Bam expression in ovaries. Normally, Bam is undetectable in the female germline until the 2-cell stage. In *nos*:Gal4 germaria there were an average of 5.2+0.2 (n = 20) Bam-negative germ cells prior to the domain of Bam detection (Figure 5A,C). In *nos>how(L)* germaria, this number had increased to 6.9+0.3(n = 18, Figure 5B,C), indicating that there were more Bam-negative germ cells in *nos>how(L)* germaria compared to control germaria (p<0.0001), therefore suggesting that Bam expression may be delayed in *nos>how(L)* germaria.

**Figure 6:**
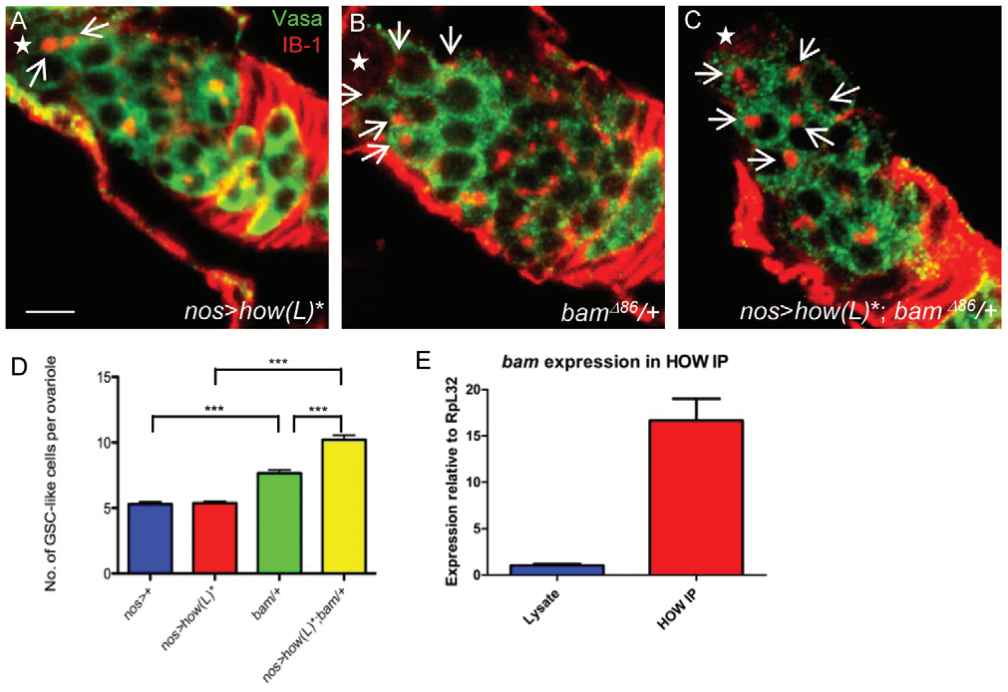
*how(L)* genetically interacts with *bam*. Comparison of the number of Vasa-positive germ cells with unbranched spectrosomes (white ↑). (**A**) Overexpression of a second, weaker *UAS:how(L)* transgene in the female germline (nos>how(L)*) does not produce germaria with extra numbers of GSC-like cells compared to wild type ovarioles. (**B**) *bam* ^Δ86^/+ germaria contain an increased number of GSC-like cells. (**C**) *nos*-driven overexpression of the weaker *UAS:how(L)* transgene in a *bam* ^Δ86^/+ mutant background results in germaria containing more GSC-like cells compared to *bam* ^Δ86^/+ ovarioles. (**D**) Graphical representation showing the mean number of GSC-like cells per germaria. (**E**) HOW immunoprecipiation from ovary lysate results in a 16.8 fold enrichment of *bam* mRNA. Anterior direction marked (*). Scale bar 5 µm.

To determine whether there was a genetic interaction between *bam* and *how* in the female germline, we asked whether overexpression of HOW(L) in the female germline could enhance the *bam/+* phenotype. As mentioned above, *bam/+* germaria were observed to contain more GSC-like cells than wild type germaria. We generated a stock which contained the *bam^Δ86^* allele and used a secondary weaker *UAS*:*how(L)* transgene, which had inserted on Chromosome II. Overexpression of this *UAS:how(L)* transgene alone in the female germline (nos>how(L)*) did not produce germaria which contained more GSC-like cells than in control (*nos>+*) germaria (5.4+0.1 n = 28, p = 0.4, Figure 6A,D), eliminating the possibility of any possible additive effects of adding the transgene to a *bam*/+ background. *bam*/+ germaria contained 7.6+0.2 (n = 30) GSC-like cells per germaria (Figure 6B,D). *nos*-driven expression of the recombined UAS:how(L)*;bam/+ strain (nos>how(L)*;bam/+) resulted in an enhancement of this phenotype, with germaria containing 10.2+0.3 (n = 32) GSC-like cells (p<0.0001, Figure 6C,D). This phenotype cannot simply be additive as nos>how(L)* germaria were normal, and suggests that *bam* and *how* genetically interact in the female germline.

We previously showed that HOW can bind *bam* mRNA in an embryonic lysate, however in order to show that HOW can similarly bind *bam* mRNA in the female germline, we used HOW antibody to immunoprecipitate HOW bound to its target mRNAs from a cell lysate comprised solely of wild type adult ovaries. After reverse-transcribing mRNA targets we amplified cDNA products using quantitative real-time PCR. We found that *bam* mRNA expression was enriched 16.8-fold (Figure 6E) in the immunoprecipitate compared to the lysate. This confirms that HOW is able to bind *bam* mRNA in germaria as well as in embryos.

## Discussion

Stem cell populations are maintained in a number of ways, but most importantly by 1) physical attachment to somatic niche cells 2) recognition of short-range proliferative signals, and 3) prevention of accumulation of differentiation-related factors. It is becoming increasingly clear that negative regulators of gene expression play an important role in maintaining many different stem cell populations by repressing the activity of differentiation factors [32], [33]. In many cases multiple regulatory mechanisms may repress a single gene and its mRNA and protein products, in order to maintain tight, developmental control over the stem cell pool while still allowing the capacity to respond to physiological cues. Here we have shown that in the female germline, as in the male germline, the RNA-binding protein HOW is required for maintenance of GSCs and exhibits genetic repression of *bam* expression.

**Figure 7:**
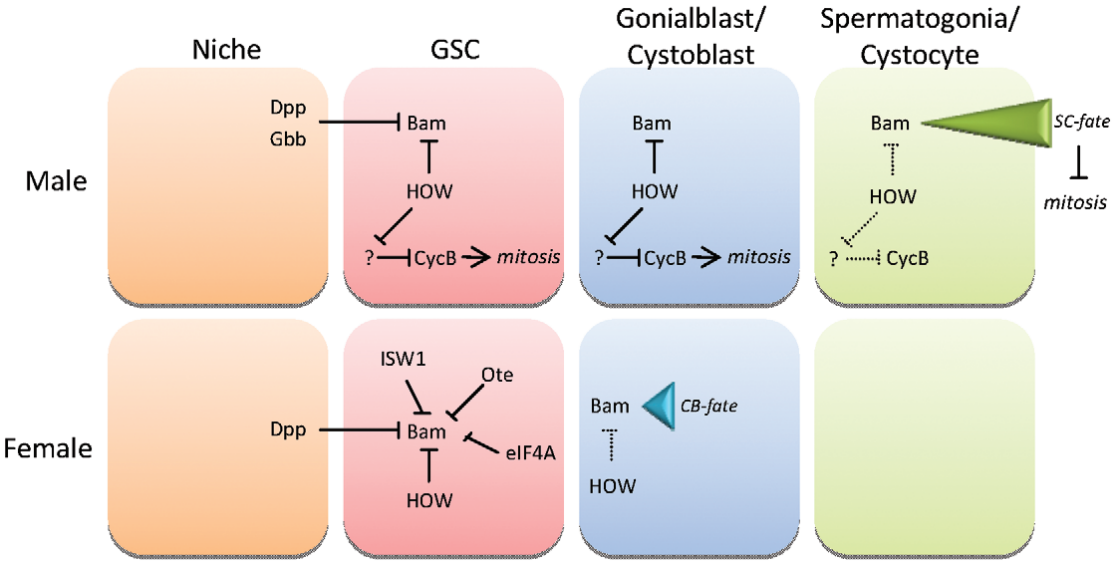
Model for HOW action in the male and female germline. In both sexes, Dpp signals from the niche or surrounding somatic cells transcriptionally repress *bam* in the GSC. In the male germline, HOW is expressed in the GSC, the gonialblast, and 2-cell spermatogonia, and is required for post-transcriptional repression of *bam* mRNA. At the 4-cell stage, levels of HOW are downregulated, coinciding with Bam protein first being detected. Bam accumulates during the spermatogonial mitotic period, and reaches a threshold value to cease mitotic amplification and initiate terminal differentiation into spermatocytes (SC). HOW also indirectly regulates CycB levels in the male germline to control the number of mitotic amplifications in which spermatogonial cells undertake prior to terminal differentiation. In the female germline, Bam is required during asymmetric stem cell division to specify the cystoblast (CB) cell fate. Levels of Bam required to initiate CB differentiation must be very low, as Bam is not observable by immunohistochemistry from the 2-cell stage. It is repressed in the GSC by many intrinsic factors including HOW. As levels of HOW are downregulated at the CB stage, Bam protein begins to accumulate, and can be visualised in 2-cell cystocytes. In the female, HOW does not play a role in regulating germ cell mitoses.

While the phenotypes that we observed when HOW levels were upregulated or downregulated in the female germline were not the same as in the male germline, these differences appear to be explained by the differential role of Bam in the two sexes (Figure 7). We previously showed that HOW binds very strongly to *bam* mRNA from *in vivo* lysates, and as *bam* contains a five nucleotide HOW recognition element [34] in its 3′-UTR, HOW was a good candidate to be a repressor of *bam* expression. One isoform of HOW, HOW(L), has been previously demonstrated as a negative regulator of target mRNAs, by binding to the 3′-UTR of its target and preventing export from the nucleus [25], [35]. As in the male germline, expression of HOW in the female germline appeared to be nuclear, indicating the prevalence of the HOW(L) isoform. Despite the expression domain of HOW being slightly more restricted in the female germline (being downregulated by the 2-cell stage compared to the 4-cell stage in males), the complementary staining pattern exhibited by Bam was also conserved in the female germline, as Bam is first detectable by immunostaining at the 2-cell stage in females. This is further indicative of HOW playing a role as a negative regulator of *bam* expression in the germline of both sexes.

Genetic evidence also suggests that HOW represses *bam* expression. Despite being termed a germ cell “differentiation factor”, Bam plays different roles in males and females. In males, Bam is required for terminal differentiation of spermatogonial cells into spermatocytes [13]. Differentiation is dependent on levels of Bam reaching a certain threshold [15]. In females, Bam is required in cystoblasts to ensure transition from the stem cell state to mitotically active cystocytes after asymmetric GSC division [16].

In both sexes, germline overexpression of HOW(L) resulted in a delay in Bam accumulation. In the male germline, some 8-cell cysts did not show detectable levels of Bam [23], while in the female germline, we observed excess early germ cells prior to the domain of Bam expression. This could be explained by the delay in *bam* expression due to increased HOW(L) expression resulting in a failure to specify cystoblast-fate during asymmetric stem cell division. As we also observed higher numbers of GSC-like cells when HOW(L) was expressed, it appears as though both possibilities may be occurring. The observation that overexpression of HOW(L) in both sexes resulted in a very similar phenotype to what has been observed in *bam* heterozygotes supports the theory that HOW represses *bam* expression. In females, *bam/+* germaria have previously been shown to contain an increased number of GSC-like cells [20], which we have also observed in this study.

The effect of losing HOW function in GSCs is also indicative of a role for HOW in regulating *bam* levels. In females, ectopic Bam expression in GSCs results in premature GSC differentiation without self renewal, whereas in males, this results in germ cell death [17], [36]. In females, *how* GSCs are lost, however, unlike in males, this may be due to premature differentiation of GSCs into cystoblasts. In females, we did not observe Bam protein in *how* mutant GSCs, however Bam is required at different levels in males and females. In the male germline, Bam gradually accumulates to an observable threshold value in order to carry out its main role, initiation of terminal differentiation of spermatogonia into spermatocytes [15]. In females, Bam is required in the cystoblast for differentiation; however Bam protein levels are undetectable at the cystoblast stage, indicating that in females, Bam can exert its effect on cystoblast differentiation at very low levels, beyond those which are observable by immunohistochemistry. As female germ cells lacking HOW were able to complete the mitotic amplification program, unlike in the male, and no *how* GSCs were observed to undergo apoptosis, it is unlikely that these cells are being lost due to cell death, suggesting that *how* GSCs are lost from the niche due to premature differentiation. Similarly, *how* GSCs were not lost as quickly from the female germline as in the male germline, which may be consistent with them surviving and differentiating as opposed to the apoptotic loss that was observed in males. While Dpp has been shown to be required for transcriptional repression of *bam* in GSCs [16], this repression may not be absolute. Chen and McKearin (2005) suggest that there are extremely low levels of Bam present in GSC spectrosomes; however they could not detect *bam* transcripts in GSCs. Therefore it is possible that a main role of HOW in female GSCs is to post-transcriptionally repress *bam* mRNA by preventing its export from the nucleus and initiating its degradation, in turn maintaining Bam protein at very low levels. Loss of HOW, therefore, would increase *bam* mRNA translation, and hence Bam protein, resulting in premature differentiation of GSCs into cystoblasts.

In females, Dpp is required in GSCs for *bam* repression [5], while in males Dpp and Gbb act cooperatively in GSCs to repress *bam* [7]. In the female germline, the short-range Dpp signal is believed to act on GSCs, but not in any cells further than one cell diameter from the niche. This could further explain why loss of HOW in GSCs does not show detectable upregulation of Bam, as *bam* is transcriptionally repressed to a large extent by Dpp and Gbb in these cells.

One feature of HOW function which has not been conserved in the female germline, is that HOW appears to have no interaction with *cycB*. In the male germline, loss of HOW led to a failure to accumulate the G2 cyclin, CycB, which is the only G2 cyclin required for mitosis in the germline [26]. The cell cycle stalled in the G2 phase of the cell cycle, cells grew abnormally large, and were removed from the germline via apoptosis. This was the prime reason for GSC loss in male *how* mutants [23]. In females, germ cells lacking HOW displayed no growth abnormalities, were able to progress through the mitotic amplification period through to the egg chamber stage, and importantly, showed no difference in CycB accumulation. As female *how* germ cells displayed no mitotic defects, this again highlights the likely possibility that GSCs lacking *how* function are lost due to premature differentiation instead of cell death, which is what was observed in male GSCs.

Therefore in the female germline, in contrast to the male, HOW does not play any role in regulating TA divisions. This suggests that the main function of HOW in the female germline is repression of *bam* mRNA as Bam is only required for cystoblast-fate specification (Figure 7). This explains why altering levels of HOW in females had no affect on TA mitoses as it does in the male.

Sex-specific germ cell cycle control has previously been demonstrated in the *Drosophila* germline. Two families which have been shown to activate the APC/C complex (targets cell cycle related proteins for degradation by the proteasome in mitosis and meiosis) are Fizzy (Fzy) and Fizzy-related (Fzr) [37]. Recently, a member of the Fzr family, Fizzy-related 2 (Fzr2) has been discovered which is detected specifically in the male germline. *fzr2* can substitute for *fzr* function when ectopically expressed in other tissue types. In *fzr* mutant embryos, there is a failure to degrade the mitotic cyclins A, B, and B3 [38], however forced Fzr2 expression can rescue this defect [39]. Fzr2 is primarily detected in pre-meiotic spermatocytes, suggesting that Fzr2 plays a meiotic role, specifically in the male germline [39]. Therefore, male-specific cell regulation of the germ cell cycle by the RNA-binding protein HOW is a possible scenario.

The RNA-binding proteins Nanos (Nos) and Pumilio (Pum) act as part of a protein complex to repress translation of *cycB* mRNA in pole cells as they migrate to the presumptive gonad during embryonic development [40]. In the female germline, Nos and Pum are expressed in GSCs and act together to prevent cystoblast differentiation [41]. The mode of action and downstream targets of this complex remain unclear but it has recently been shown that in the cystoblast, Nos is post-transcriptionally repressed by Bam [42], allowing expression of differentiation genes. Pum, despite its role in the maintaining proliferative GSCs [41], [43], plays a different role in the cystoblast. Pum functions together with a potent differentiation gene, *brain tumor*, to repress self-renewal targets such as *Mad* and *dMyc* [44]. While Nos expression is present in the male germline, Pum is expressed at very low levels (or essentially not at all) in the male. While a number of Pum mutant alleles exhibit female sterility [41], [43] no functional role for Pum has been demonstrated in the male germline. This is another example of differential use of RNA-binding proteins in the male and female germline.

The exact mechanism by which HOW regulates *cycB* in the male germline is yet to be elucidated, but since loss of HOW function results in loss of *cycB* expression it could be predicted that HOW is repressing a negative regulator of *cycB* expression.

The role of HOW in regulation of Bam appears to have been conserved between the sexes. It is now apparent that *bam* expression is regulated at various levels (see below). As reproduction is of critical importance for the survival of the species, it is not surprising that key regulators of this process have evolved tight controls on their expression and function. Proper expression of Bam is vital for maintaining tissue homeostasis, and misexpression has serious outcomes. *bam* has previously been shown to be transcriptionally repressed by the Dpp pathway in both sexes [5], [7], however recent studies have also shown that a number of other factors, such as ISW1 [18], Otefin [19], EIF4A [20], and Piwi [21] also play various roles in maintaining *bam* repression in GSCs. Here we show that HOW is responsible for *bam* mRNA regulation at a post-transcriptional level in both sexes. This complex and redundant regulation of stem cell proliferation highlights how important tight control of stem cell behavior is for the organism.

## Materials and Methods

### Cytology

Ovaries were fixed and immunostained as per [45]. Serial confocal sections were taken on a Ziess LSM510 Confocal Microscope. Ovaries were immunostained with 10 µg/ml 4,6-diamidino-2-phenylindole (DAPI, Sigma), 1∶50 rabbit anti-HOW (T.Volk), 1∶500 rabbit anti-GFP (Molecular Probes), 1∶500 mouse anti-GFP (Invitrogen), 1∶100 goat anti-Vasa (Santa Cruz), 1∶50 mouse anti-Fasiclin3 (Developmental Studies Hybridoma Bank, DSHB), 1∶25 mouse anti-Bam-S (DSHB), 1∶50 mouse anti-α-IB-1 (DSHB), 1∶100 mouse anti-Fibrillarin (Abcam), 1∶500 rabbit anti-CycB [46], 1∶50 mouse anti-HA (Cell Signaling Technologies). ApopTag staining was performed using Chemicon Kit and procedures followed from manufacturer's instructions.

### Detection of HOW target mRNAs

Adult ovaries were homogenised by grinding gently in 150 µl polysome lysis buffer containing 0.5% Triton X100 supplemented with 1 mM Dithiothreitol,10 µl/ml ProtoCEASE™ protease inhibitor (G-Biosciences, St Louis MO USA) and 100 units/ml RNasin™ (Promega Madison, WI USA). Homogenate was sonicated to disrupt nuclear membranes, lysate centrifuged and supernatant incubated overnight at 4°C with anti-HOW antibody coated Protein A Dynabeads™ magnetic beads prepared according to the manufacturer's instructions (Invitrogen, Carlsbad Ca USA). Following incubation the bead-Ab-Ag complex was washed in buffer containing protease and RNA inhibitors and resuspended in TES buffer (10 mM TrisHCL pH 7.5 1 mM EDTA 1% SDS) prior to RNA elution and quantification. Quantitative PCR conditions were optimised and target specificity confirmed using cDNA prepared from embryo lysate mRNA. HOW-bound RNA was eluted from beads and collected in DEPC water prior to reverse transcription and analysis by quantitative real time PCR with an Opticon 2 real-time thermocycler (Bio-Rad Hercules, CA USA).

Real time oligonucleotide primers were designed for *bam* (forward 5′- GCGCTCGCGCCATTTTGCAT-3′and reverse 3′-TATCCGCGGACGCAGAGCCT-5). Gene expression was normalised to the housekeeping gene RpL32, using oligonucleotides (forward 5′-ATCGGTTACGGATCGAACAA-3′ and 3′-TGGGCGATCTCGCCGCAGTA-5′). Primer efficiencies of Bam and String versus Rpl32 were confirmed as being similar by correlation analysis of cycle threshold (Ct) values of 10-fold dilutions of embryo cDNA. For expression analysis, data from 3 replicates were analyzed using the 2^-ΔΔC^_T_ method.

### Statistics

Statistical analyses were performed using Graphpad Prism and reported as mean + standard error of the mean. P-values were obtained by Student's t test.

### Generation of marked clones

GFP-negatively marked homozygous clones were generated using the heat-shock inducible Flp-FRT system. *hs*-FLP/Y; FRT82B *how* ^stru^/FRT82B *Ubi*-GFP or *hs*-FLP/Y; FRT82B/FRT82B *Ubi*-GFP adult males were heat shocked at 37°C twice for 1 hour (18 hour interval) and raised at 25°C for the appropriate length of time. Control twinspot clones were counted after immunostaining for CycB.

### Fly strains

Fly strains used for this study include w^1118^, *bam*-GFP [27], *nos:Gal4* [29], FRT82B *how^stru-3R-3^* (Bloomington *Drosophila* Stock Center, BDSC), *hs*-FLP; FRT82B, *Ubi*-GFP (BDSC), P(lacW)*how* ^j5B5^/TM3 (BDSC), *UAS:HOW(L)* [25], *bam^Δ^* ^86^ (*Drosophila* Genetic Resource Center, Kyoto). Flies were raised at 29°C to maximise GAL4 activity.
